# Emerging Nonlinear Photocurrents in Lead Halide Perovskites for Spintronics

**DOI:** 10.3390/ma17081820

**Published:** 2024-04-16

**Authors:** Jianbin Chen, Hacer Koc, Shengkai Zhao, Kaiyu Wang, Lingfeng Chao, Mustafa Eginligil

**Affiliations:** Key Laboratory of Flexible Electronics (KLoFE) and Institute of Advanced Materials (IAM), School of Flexible Electronics (Future Technologies), Nanjing Tech University, 30 South Puzhu Road, Nanjing 211816, China; 18258857615@163.com (J.C.); haccerkoc@gmail.com (H.K.); zhaoshengkai4@gmail.com (S.Z.); 202161122152@njtech.edu.cn (K.W.)

**Keywords:** nonlinear photocurrent, Rashba effect, Rashba-Edelstein effect, spintronics, circular photogalvanic effect, photo-induced inverse spin Hall effect, lead halide perovskites

## Abstract

Lead halide perovskites (LHPs) containing organic parts are emerging optoelectronic materials with a wide range of applications thanks to their high optical absorption, carrier mobility, and easy preparation methods. They possess spin-dependent properties, such as strong spin–orbit coupling (SOC), and are promising for spintronics. The Rashba effect in LHPs can be manipulated by a magnetic field and a polarized light field. Considering the surfaces and interfaces of LHPs, light polarization-dependent optoelectronics of LHPs has attracted attention, especially in terms of spin-dependent photocurrents (SDPs). Currently, there are intense efforts being made in the identification and separation of SDPs and spin-to-charge interconversion in LHP. Here, we provide a comprehensive review of second-order nonlinear photocurrents in LHP in regard to spintronics. First, a detailed background on Rashba SOC and its related effects (including the inverse Rashba–Edelstein effect) is given. Subsequently, nonlinear photo-induced effects leading to SDPs are presented. Then, SDPs due to the photo-induced inverse spin Hall effect and the circular photogalvanic effect, together with photocurrent due to the photon drag effect, are compared. This is followed by the main focus of nonlinear photocurrents in LHPs containing organic parts, starting from fundamentals related to spin-dependent optoelectronics. Finally, we conclude with a brief summary and future prospects.

## 1. Introduction

As the effect of charge properties of electrons on electronic devices is limited by Moore’s law, the future of electronics, information processing, and storage has to rely on alternative methods. One of the most promising methods is spintronics, which emerged several decades ago and concerns the functionalization of spin properties of electrons [1]. Spintronics has rigorous physical foundations, material aspects, and control methods to manipulate the spin-dependent electronic properties of materials, such as the conductivity of materials—metals, semimetals, or semiconductors; dimensionality and flexibility; and magneto-electrical or optoelectronic routes. As a magneto-electrical transport phenomenon, the giant magnetoresistance (GMR) effect in ferromagnetic metals [2] and perovskite-like ceramics [3] was an important step forward for spintronics. GMR and similar effects concern controlling the movement of charge carriers, induced by their spin, in an electronically active layer, which can be also an organic semiconductor [4] and can use the advantage of being flexible. Although magnetic semiconductors play a decisive role [5] in the era of GMR studies and magneto-resistive random-access memories based on the tunneling effect [6] are already in use in the information storage industry, the dissipation and retrieval of spin information are major challenges.

The ultimate goal of spintronics is to achieve reliable spin-dependent currents, i.e., spin information transfer from one point to another with a minimum loss, and to obtain information processing via switching mechanisms simultaneously with information storage. In both processes, bulky materials of 3D would be the reason for higher spin information loss, whereas materials with lower dimensions of 1D and 2D, confined structures, hetero-interfaces, or so-called low-dimensional systems with strong spin–orbit coupling (SOC) are expected to be better candidates to make spintronics work by meeting certain necessary criteria, such as high carrier mobility, high surface area, and low resistivity. Recently, large MR has been observed in a layered magnetic semiconductor [7] and a few nm thick III–V semiconductors influenced by the magnetic proximity effect [8] but at low carrier densities, which exemplify the shortcomings of magnetic semiconductors. In this context, among strong SOC low-dimensional materials, 2D materials and hybrid organic–inorganic perovskites (HOIPs) stand as the most prominent ones for spintronic devices [9]. For example, 2D materials have been used in the fabrication of spin memory devices and spin transistors with low power consumption and high-speed operation with well-maintained spin information [10]. At the same time, large unsaturated MR in semimetal 2D layered WTe2 [11] demonstrated a perfect charge carrier (electron–hole) balance, indicating highly sensitive carrier tunability and band topology, allowing symmetry breaking-induced spin polarization generation without an applied magnetic field [12]. On the other hand, the flexibility of semiconducting HOIPs in terms of material and device preparation as well as band gap and SOC tunability has been as attractive in 2D materials for spin injection and manipulation [9] with the advantages of opto-spintronic properties [13].

Excitation by polarized light is quite attractive in HOIP spintronic studies; for instance, it was possible to generate right- and left-circularly polarized light states using ferromagnetic (FM) electrodes on HOIP and inject spin-polarized carriers, and consequently, a spin light-emitting diode (LED) was realized [14]. The opposite action of a spin LED, i.e., conversion of polarized light into spin-dependent optoelectronic signals, does not require FM electrodes in HOIPs thanks to its inherently strong SOC and the Rashba effect [15]. These optoelectronic signals can be simply generalized as a light helicity-dependent photocurrent (LHDP) taking place due to the light–matter interaction for a given crystal symmetry in any material system and were studied experimentally in II–VI and III–V quantum wells more than two decades ago [16]. Furthermore, the phenomenology of polarized light interacting with various materials with different microscopic mechanisms and optical selection rules is now at a satisfactory level of understanding. Additionally, detailed LHDP studies on graphene [17], topological insulators [18], and topological semimetals [19] in infrared and terahertz excitation regimes are promising for next-generation optoelectronics. Furthermore, LHDP on genuine 2D semiconductors [20] and emerging HOIPs in the visible region [21] have been elaborated on and photocurrents due to the circular photogalvanic effect based on noncentrosymmetry have been identified.

Among the pool of materials, HOIPs have become more appealing in terms of spin-dependent LHDP since at the interfaces and/or surfaces, their unique optoelectronic properties make them ideal opto-spintronic and spin–orbitronic materials [22]. Here, we present a comprehensive review of the nonlinear photocurrent in the most well-known HOIPs, lead halide perovskites (LHPs), containing organic parts from the spintronic point of view and excluding chiral perovskites. This approach is applicable to all LHPs and also all metal halide perovskites. By starting with the Rashba SOC and the inverse Rashba–Edelstein effect (IREE), necessary knowledge of the phenomenology of the photocurrent (or photovoltage) nonlinear-in-electromagnetic field with a focus on the most important LHDP in terms of spintronics, namely the circular photogalvanic effect (CPGE), will be introduced with examples of model semiconductor systems in the literature. Then, the photo-induced inverse spin Hall effect (PISHE), which shares the same phenomenology as the CPGE, will be compared with the CPGE, and their features that are distinct from the bulk effects will also be provided in the context of metal and semiconductor behavior. This will be followed by a brief background on LHPs related to spintronic and nonlinear photocurrent, particularly the CPGE both in a single crystal and polycrystalline LHP. Spin-to-charge conversion processes by IREE and PISHE in LHP will also be discussed. Finally, a conclusion together with future prospects will be given.

## 2. Spin–Orbit Coupling and Symmetry Consideration

### 2.1. Spin–Orbit Coupling and Rashba Effect

Approximately 70 years ago, it was accepted that an electron’s spin in an orbit inside atoms is coupled to their orbital movement, known as spin–orbit coupling (SOC), and it can have important consequences for solid-state physics [23]. The SOC can be seen as an analog of Zeeman energy for an electron with mass m, momentum **p** (wave vector **k**) moving in an electric field **E**, with Hamiltonian Ĥ_SO_~µ_B_ (**E** × **p**)/mc^2^, where µ_B_ is the Bohr magneton and c is the speed of light; it has time-reversal symmetry and requires broken spatial inversion symmetry [24]. The major outcome of SOC is the spin splitting of semiconductor bands, which were studied in inversion asymmetric zincblende structures with pronounced additional cubic dispersion by Dresselhaus [25] and inversion asymmetric wurtzite structures with leading linear dispersion by Rashba and Sheka [26]. In 1984, Bychkov and Rashba introduced a simple expression of SOC to explain the properties of electron spin resonance in 2D electron gas systems with C_2v_ point group symmetry and inversion asymmetry along the growth direction [27], and this coupling with linear dependence in the wave vector, which can be modified by external fields, was named Rashba SOC since the original discovery was made back in 1959 for electron systems by Rashba himself (see review [28]). In the last few decades, the Rashba SOC and the Rashba effect have inspired a large number of predictions, discoveries, and novel concepts far beyond semiconductors, mostly concerning 2D electron systems, including interfaces and surfaces and the surface states in a new class of matter, namely topological insulators [29]. There is growing interest in the Rashba SOC in 2D materials, such as transition metal dichalcogenides and perovskites [30,31]; in particular, the possibility of spin manipulation of electrons by an applied electric field was extremely attractive for spintronic applications.

**Figure 1:**
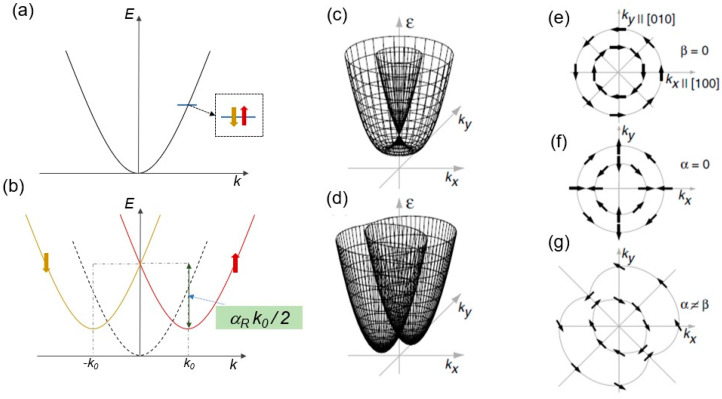
Description of the Rashba effect. (**a**) Parabolic conduction band (CB) with spin-up and spin-down electrons occupying the same energy state and the CB minimum is at momentum *k* = 0. (**b**) The CB is split by the Rashba effect, by *k* = *k*_0_ corresponding to an energy shift (α_R_ *k*_0_/2) due to Rashba spin–orbit coupling (SOC) strength (α_R_); one band with spin-up (with minimum at *k*_0_), the other with spin-down (with minimum at −*k*_0_). (**c**–**g**) Schematics of 2D band structure with wave vector *k*-linear terms for C_2v_ symmetry for different relative strengths of structural- and bulk-inversion asymmetry (SIA and BIA). (**c**) The distribution of spin orientations on the 2D Fermi surface is shown for the case of only Rashba or Dresselhaus SOC alone, while (**d**) shows the case of coexistence. (**e**,**f**) 2D plots for the Rashba SIA (α) and the Dresselhaus BIA (*β*), respectively (arrows indicate the spin orientations); note that spin directions are opposite from +*k*_x_ to −*k*_y_ (same in ±*k*_y_) in either case. While the spin orientation is always tangential in the case of SIA, it is perpendicular in the case of BIA at ±*k*_x_ and ±*k*_y_ and tangential otherwise. (**g**) 2D plot of the coexistence case of (**d**). Adapted with permission from [35]. © 2004 American Physical Society https://doi.org/10.1103/PhysRevLett.92.256601.

Figure 1a,b depicts a simple understanding of the Rashba effect. A parabolic energy band in Figure 1a, with its minimum at wave vector k = 0, is spin-degenerate. Electrons with both spin-up and -down can have energies E± = (ħ^2^ **k**^2^)/2m* at ±k wave vector values, where ħ is the Planck’s constant and m* is the effective mass of electrons. This band can be split into two spin-dependent bands, as in Figure 1b, due to the Rashba effect. After band splitting, the minima of these two bands are now located at k = ±k_0_. With respect to k = 0 of the band in the absence of the Rashba effect, the minima of these two bands are shifted by an energy ± α_R_ k_0_/2, where α_R_ is the Rashba SOC coefficient.

### 2.2. Rashba Effect vs. Dresselhaus Effect

The Rashba effect depicted in Figure 1a,b is the simple description of SOC (*k*-linear), and in fact, if there is strain in the (001) direction, SOC due to the Dresselhaus effect is also reduced to the same representation (from *k*-cubic to *k*-linear). The Rashba effect for 2D electron–hole systems can be different (see [32] for details). Furthermore, dimensionality and carrier type and density can also be crucial. For instance, in 3D electron systems (high carrier density), the *k*-cubic Dresselhaus term leads, while in 3D hole and 2D electron and hole systems with low carrier densities, the *k*-linear Dresselhaus term dominates. While the *k*-linear Rashba term is expected to be absent in a 2D hole system (the case of excited bulk conduction bands is not included), the k-linear and -cubic Dresselhaus terms are comparable in inversion layers of GaAs [33]. Also, the relationship between the Rashba and Dresselhaus effects could be intricate with varying physical parameters and applied fields. For example, for an in-plane applied magnetic field larger than the SOC, quantum corrections are determined by the *k*-cubic Dresselhaus term, while the linear Rashba and Dresselhaus terms cancel each other [34]. Nevertheless, simply, both effects leading to SOC can be observed in semiconductor heterostructures due to the asymmetry of a quantum well, interface, or surface, such as structural-inversion asymmetry (SIA) as the Rashba effect and bulk-inversion asymmetry (BIA) underlying the bulk crystal structure and interface-inversion asymmetry (IIA) as the Dresselhaus effect, with BIA and IIA being inseparable [35], where IIA lies somewhere between SIA and BIA [36]. While α = α_R_ is the Rashba coefficient responsible for SIA, β is the Dresselhaus coefficient for the combination of BIA and IIA.

The case of [27] can be observed in zincblende-based quantum well (QW) structures with SIA along the growth direction and the point group symmetry reduced to C_2v_, such as in InAs QWs with 2D electron gas [35], where SIA can be varied by delta doping and may coexist with BIA. Indeed, D_2d_, C_2v_, and C_s_ are the main point group symmetries for zincblende-based QWs [37]. In the case of QWs grown along (001), when the symmetry is D_2d_, only BIA can exist. With the addition of interface effects such as asymmetric doping, D_2d_ can be reduced to C_2v_, which makes the strength of SIA comparable to BIA, and further asymmetry by heterojunctions leads to SIA dominating [16]. For SIA (Rashba) and BIA + IIA (Dresselhaus), Hamiltonians can be expressed as Ĥ_SIA_~*β^a^*_xy_ (*σ*_x_*k*_y_ − *σ*_y_*k*_x_) and Ĥ_BIA+IIA_~*β^s^*_xy_ (*σ*_x_*k*_y_ + *σ*_y_*k*_x_), where *β^a^*_xy_ and *β^s^*_xy_ are antisymmetric and symmetric components of pseudo-tensor *β*_lm_ in the (*x*,*y*,*z*) coordinate system, and *σ*_l_ is the Pauli matrice. Taking the C_2v_ symmetry as an example, the pseudo-tensor *β*_lm_ has two integrable components, α = *β*_xy_ = −*β*_yx_ and *β* = *β*_xx_ = −*β*_yy_, yielding the Rashba and Dresselhaus terms, respectively. Figure 1c plots energy as a function of *k*_x_ and *k*_y_ either for only SIA (Figure 1e) or only BIA (Figure 1f). Assuming that both are nonzero, the cases in which the intensities of SIA and BIA are (not) equal are also shown in Figure 1f,g, with spin orientations. This exemplifies the competition between the Rashba and Dresselhaus terms and it is applicable to other symmetry groups. One important way to identify these two contributions is nonlinear photocurrent spectroscopy, which will be shown later [35].

### 2.3. Rashba-Edelstein Effect

Geometries of low-dimensional systems such as interfaces and surfaces can result in symmetry reduction, as mentioned above. One important geometric effect for lattices is inversion symmetry, which can be broken by placing a material onto another or at the interface where two different materials meet. Broken inversion symmetry has several consequences in terms of spin-related phenomena, such as the Edelstein effect (EE), also known as current-induced spin polarization (also known as the inverse spin-galvanic effect) [38]. In a nonmagnetic system with broken inversion symmetry, an applied electric field can produce homogeneous spin polarization in a direction normal to the applied field. In this sense, the EE is similar to the spin Hall effect (SHE), where a longitudinal charge current is accompanied by a transversal pure spin current (or voltage) [39,40]. The phenomenology behind the EE and SHE is similar; however, their physical origin is different, as the SHE is rather a bulk effect. The EE can be thought of as charge-to-spin conversion, while inverse EE (IEE) is spin-to-charge conversion, and it is possible to observe EE or IEE for several material systems [41].

**Figure 2:**
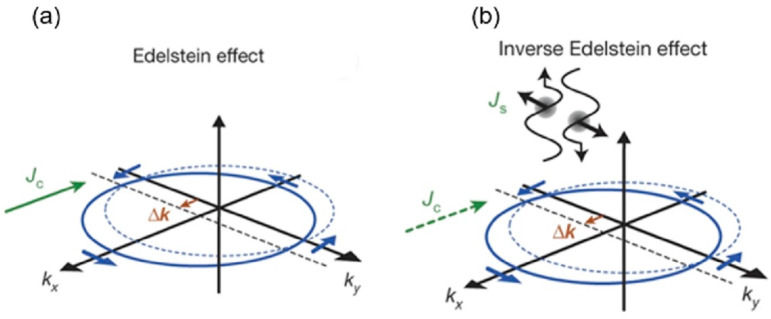
(**a**) *k*-space schematic of the Edelstein effect. A charge current *J*_c_ at the surface causes a shift Δ***k*** of the Fermi contour, resulting in a non-zero spin density, leading to a spin current. (**b**) *k*-space schematic of the inverse Edelstein effect. Injecting a spin current (*J*_s_; spin-polarized wiggles) leads to an imbalance of the spin state occupation, generating a charge current (From [43]).

On the other hand, as EE and IEE are related to Rashba interfaces and surfaces, they are also called the Rashba–Edelstein effect (REE) and the inverse Rashba–Edelstein effect (IREE), as observed at the interfaces of nonmagnetic materials [42]. SOC is essential in low-dimensional systems for the observation of the REE and IREE. REE refers to the lateral displacement of spin-polarized electrons in a material in the presence of SOC, which results in spin splitting. In a Rashba system, the momentum and spin degrees of freedom are locked. A charge current *J*_c_ at the surface, along the *x*-direction, induces a shift Δ*k* of the Fermi contour, resulting in net spin polarization along the contour, which eventually turns into a spin current along the *y*-direction in the conducting material at the other side of the interface; thus, it is considered charge-to-spin conversion, or the REE (Figure 2a). On the other hand, the opposite effect of spin-to-charge conversion by the IREE takes place upon injecting a spin current along the *y*-direction (*J*_s_; spin-polarized “wiggles” [43]), as seen in Figure 2b, which leads to an imbalance of occupied spin states on one side of the Fermi contour and generates a net charge current along the *x*-direction in the conducting material on the other side of the interface. Simply, the REE is an effect that generates a pure spin current, while the IREE is an effect that converts spin current into charge current. This offers the possibility to manipulate electron spin—one of the main goals of spintronics. Although the Rashba-related effects, namely SOC, REE, and IREE, have been predominantly studied theoretically and experimentally in topological insulators’ surfaces and interfaces [24,43], there has been a surge of interest in Rashba-related effects in HOIPs [44]. Note that there is a large Rashba SOC in LHPs, such as CH_3_NH_3_PbI_3_ with C_4v_ symmetry [45]. Also, the observation of the IREE due to the Rashba interface in CH_3_NH_3_PbBr_3_ [46], with a long spin relaxation time of up to ~190 ps in single-crystal samples [47], was an important step for organic spintronics.

## 3. Nonlinear Photo-Induced Effects

The interaction of an electromagnetic (EM) wave with matter can be linear or nonlinear. The polarization state of a light field (or only the electric field of the EM wave for a nonmagnetic medium) can be varied by optical instruments, such as wave retarders; then, light with a certain polarization state can impinge on the surface of a medium and change its polarization. The polarization vector of light ***P*** and the polarizability of the medium has a relationship, which involves the electric field of light ***E*** and the polarizability tensor *χ*. The electric field ***E*** has the form:

(1) $$E\left(r,t\right)=E\left(\omega ,q\right)e^{i\left(qr-\omega t\right)}+E^{*}\left(\omega ,q\right)e^{-i\left(qr-\omega t\right)},$$

where ***r*** and *t* are the positions of the plane wave and ***q*** and *ω* are the wave vector and frequency of the light. The relationship between the polarizability tensor χ and the polarization vector ***P*** can be simply given as

(2) $$P=\varepsilon_{0}\;\left(\chi^{\left(1\right)}E+\chi^{\left(2\right)}\;EE\;+\ldots \right),$$

where $\varepsilon_{0}$ is the vacuum permittivity and χ^(n)^ is the n^th^ order polarizability tensor, for n = 1, 2, and so on (only shown up to 2nd order).

In Equation (2), the first term is linear in ***E***, and $\chi^{\left(1\right)}$ is known as the linear polarizability term. The second term is the vector multiplication of the ***E*** field of Equation (1), and $\chi^{\left(2\right)}$ is the second-order nonlinear polarizability tensor, which contains two terms, time-dependent *ac* and time-independent *dc* terms. There are several common nonlinear optoelectronic effects of the second order. The time-dependent second-order nonlinear response is known as second harmonic generation (SHG). It refers to the shortening of the wavelength by half and the doubling of the energy when the light beam passes through a noncentrosymmetric medium. Here, the SHG can be taken as χ2′EEe2iqr−ωt+χ2′E**E*e−2iqr−ωt, where $\chi^{\left(2^{\prime }\right)}$ is the second-order time-dependent polarizability tensor, where ′ is the symbol for time dependence and * is the complex conjugate. Doubling the frequency, the SHG, is a widely used technique to achieve UV generation. This polarizability does not contribute as a nonlinear photocurrent.

In the case of two-photon processes, the incident light will have two frequencies, *ω*_1_ and *ω*_2_. Then, the electric field can be simply expressed as $E=E_{1}\;cos\omega_{1}t+E_{2}\;cos\omega_{2}t$. Substituting the second-order term yields the two-photon SHG as $P^{\left(2\right)}=\varepsilon_{0}\;\chi^{\left(2\right)}\;{\left(E_{1}\;cos\omega_{1}t+E_{2}\;cos\omega_{2}t\right)}^{2}$, which can have a contribution as a nonlinear potential difference (not a photocurrent). Among the other nonlinear photo-induced effects, the Kerr effect should be mentioned, which is a change in the refractive index of a material under a strong light field—useful when making optical switches and modulators. There is also a self-focusing effect of the beam due to the nonlinear refractive index during the propagation of the light beam, which is useful when manufacturing micro-opto-mechanical systems.

Nonlinear photo-induced effects have direct applications in optical communication, computing, and data storage, mainly due to their transport features (photocurrent), particularly the ones due to an electron’s spin and spin–orbit interaction in materials with certain symmetry arrangements. Here, we focus on the second-order time-independent term (the *dc* term) of Equation (2), which is of the form of $\chi^{\left(2\right)}EE^{*}$. This is the source of nonlinear photocurrent and photovoltage.

### 3.1. Spin-Related Nonlinear Photocurrents at Oblique Incidence

Spin photocurrents, due to nonlinear photo-induced effects, have a history of more than 50 years. In recent years, as novel low-dimensional materials have been experimentally realized, theories and experiments related to spin-related nonlinear photocurrents have gained popularity, e.g., II–VI and III–V semiconductor quantum wells [16]. Such a spin photocurrent can be described as the conversion of the angular momenta (spin, orbital, or both) carried by photons, depending on optical selection rules, to vary the spin states of electrons in a material and read it as a current (spin or charge). The generation of these spin photocurrents is possible using two main mechanisms: either by direct photoexcitation (the generation of electron–holes in a direct bandgap semiconductor) or scattering of photogenerated carriers (involving phonons). In the former case, the generation of spin currents can be taken as being due to the intrinsic optical response; in the latter, it is due to extrinsic effects such as impurities within the material being probed by polarized light. In either case, the polarized light impinging on the sample surface at a certain angle of incidence (an oblique angle) forces electrons to obey the spin-dependent optical transition rules (either interband or intraband). As a result, the electrons with particular spins will move in a certain direction, yielding a net current. Simply, the amplitude and direction of this spin photocurrent can be controlled by the helicity of the excitation, and the direction of photocurrents will be opposite for left- (σ−) and right- (σ+) circularly polarized excitations, which can yield a circular photocurrent.

#### 3.1.1. Phenomenology

In the context of spin-related photocurrents or any second-order photocurrent, the time-independent *dc* term in Equation (2), $\chi^{\left(2\right)}EE^{*}$, should be paid more attention. We should remind the reader that $\chi^{\left(2\right)}$ can be a third- or fourth-rank tensor, $\sigma_{\alpha \beta \gamma }^{\left(2\right)}$ or $\xi_{\alpha \beta \gamma \delta }^{\left(2\right)}$, and the current associated with it can be expressed as

(3) $$j_{\alpha }=\sigma_{\alpha \beta \gamma }^{\left(2\right)}E_{\beta }E_{\gamma }^{*}+\xi_{\alpha \beta \gamma \delta }^{\left(2\right)}q_{\delta }E_{\beta }E_{\gamma }^{*},$$

where $q_{\delta }$ is light momentum, and *α*, *β*, *γ*, and *δ* are the indices that could be *x*, *y*, or *z*. The first term in Equation (3) does not contain light momentum and has a third-rank conductivity tensor, while the second term contains light momentum and has a fourth-rank conductivity tensor.

The phenomenology of a second-order nonlinear response to light passing through a quarter wave plate (QWP) and incident on a sample plane with *θ* and *ζ* incidence and azimuthal angles, respectively, can be expressed according to the Jones calculus [48]. The transmitted electric field $E^{\prime }$ can be written in terms of *E*_0_ (the magnitude of the electric field just before entering the QWP) as

(4) $$E^{\prime }=E_{0}\;\left(\begin{array}{c} s\;\sin\zeta +p\;\cos\zeta \;\cos\theta \\ -s\;\cos\zeta +p\;\sin\zeta \;\cos\theta \\ p\;\sin\theta \end{array}\right)e^{i\left(qr-\omega t\right)},$$

where $s=i\;\left(\sin2\varphi \right)/\sqrt{2}$ and $p=\left(i\;\left(\cos2\varphi \right)-1\right)/\sqrt{2}$. From here, we can see that $E_{\beta }E_{\gamma }^{*}$ can only depend on $s^{2}$, sp, and $p^{2}$. Therefore, $j_{\alpha }$ can only depend on isin2φ, sin4φ, cos4φ, icos2φ, and a constant. However, for ζ=nπ/2, icos2φ should vanish.

Then, the polarization-dependent photocurrent $j_{\alpha }$ with conductivity tensors of either $\sigma_{\alpha \beta \gamma }^{\left(2\right)}$ or $\xi_{\alpha \beta \gamma \delta }^{\left(2\right)}$ can be simplified as

(5) $$j_{\alpha }=C_{1}\sin\left(2\varphi \right)+L_{1}\sin\left(4\varphi \right)+L_{2}\cos\left(4\varphi \right)+D,$$

where the constants $C_{1}$, $L_{1}$, $L_{2}$, and D are polarization-dependent photocurrent coefficients depending on the way they are related to φ, except D, which does not depend on φ. There is also a cos2φ term, $C_{2}$, which is usually thought to be similar to $C_{1}$, accepted as a deviation from homogeneity. Nevertheless, regardless of the specific behavior of all terms, φ is the most crucial experimental parameter in simple phenomenology. Here, the $\sin\left(2\varphi \right)$ term includes, exclusively, photocurrents due to the circular polarization state of light, while photocurrents with $\sin\left(4\varphi \right)$ and $\cos\left(4\varphi \right)$ dependencies originate from the linearly polarized light. The terms $C_{1}$, $L_{1}$, $L_{2}$, and D include angle of incidence dependence and the existence of light momentum transfer or not.

#### 3.1.2. Photogalvanic and Photon Drag Effects

If there is no light momentum transfer, the first term of Equation (3) is valid, known as the photogalvanic effect (PGE) and if there is light momentum transfer, the second term of Equation (3) is valid, known as the photon drag effect (PDE). Considering that the $C_{1}$ term is related to the circularly polarized excitation, the first term of Equation (3) can be taken as circular PGE or CPGE, while the second term of Equation (3) can be taken as circular PDE or CPDE. Although the phenomenology was defined long ago, the microscopic theories of photocurrent due to the CPGE and the CPDE for various physical systems have been developed in recent years.

The symmetric part of $\sigma_{\alpha \beta \gamma }^{\left(2\right)}$ in Equation (3) can describe the linear polarization dependent part, the so-called linear PGE (LPGE), which is a combination of $L_{1}$ and $L_{2}$ terms of Equation (5). $j_{\alpha }$ is odd under time reversal, which means it can change sign when the wave vector ***k*** changes sign. However, the products and combinations of $E_{\beta }E_{\gamma }^{*}$ can be either even or odd under time reversal, antisymmetric or symmetric, respectively. When this product is even or antisymmetric and even ***k*** changes sign, $j_{\alpha }$ will not change signs. This corresponds to the CPGE, which is exclusively related to circularly polarized excitation. The main difference between the LPGE and the CPGE is that the LPGE is only allowed in noncentrosymmetric media, while the CPGE is allowed in centrosymmetric media as well for reduced symmetry at low dimensions, such as edges, interfaces, heterostructures, and few-layer materials. The light propagation in that medium is gyrotropic in nature and a difference in photo-induced currents can take place between right and left circular polarized excitation. In quantum wells, such currents induced by the CPGE are included in the category of spin photocurrents along with the spin-galvanic photocurrents. While the latter do not require polarized light and can take place in an applied magnetic field, the CPGE can fall into the category of pure spin photocurrents. The LPGE is an effect independent of photon helicity, and its origin is due to the scattering asymmetry of free carriers. It is an intrinsic property of the material itself and can be a measure of anisotropy. It is not possible to talk about spin photocurrents in the case of the LPGE.

As PDE has light momentum dependence, unlike the PGE, it can also take place in inversion symmetric systems. Similar to the PGE, symmetric and antisymmetric parts of the conductivity sensor $\xi_{\alpha \beta \gamma \delta }^{\left(2\right)}$ of Equation (3) yield linear PDE (LPDE) and CPDE, respectively. Again, $j_{\alpha }$ is odd under time reversal, but since light momentum is also odd, the products of $E_{\beta }E_{\gamma }$ and the tensors associated can be odd or even, respectively. That makes the major difference between the PGE and the PDE the time reversal. As the PDE is a bulk effect, it is not allowed at normal incidence, while the PGE is possible at normal incidence and can be nonzero at the edge or interface where there could be a reduction in symmetry in replacement of noncentrosymmetry. As in the case of the LPGE, the current direction and magnitude due to the LPDE are independent of the photon helicity and are not symmetry-limited.

#### 3.1.3. Influence of Symmetry and Excitation on CPGE

**Figure 3:**
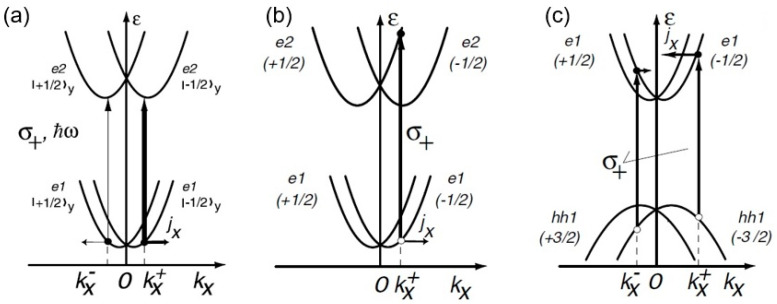
Microscopic mechanisms of CPGE for (**a**) C_2v_ intraband, (**b**) C_s_ intraband, and (**c**) C_s_ interband excitations (only shows σ+) leading to photocurrent *j*. The thickness difference in vertical arrows in (**a**) represents spin-conserving transitions (such as from e1 (−1/2 spin) conduction band state to e2 (1/2 spin)) in C_2v_, while there is spin-flipping in C_s_ (as shown in (**b**)). Spin splitting together with interband optical selection rules shown for C_s_ in (**c**) result in occupation imbalance of the positive and negative states, yielding a spin-polarized photocurrent (From [16]).

There could be various microscopic mechanisms behind the CPGE based on point group symmetry and the excitation wavelength. For instance, in n-type QWs with C_2v_ symmetry (involving spin-conserving optical transitions) [37], there could be direct intraband transitions within the CB, as described in Figure 3a. For given light polarization (say σ+), the transition rate for electrons with the opposite spin orientation for an in-plane direction of light propagation will differ (indicated by the thickness difference in two vertical arrows) and lead to an asymmetrical distribution of carriers in ***k***-space and a net photocurrent [16].

In the case of reduced symmetry of C_s_ in n-type QWs, there are spin-flip processes and transitions from the lower split-bands to upper split bands that will add up for a given polarization state (such as σ+), as shown in Figure 3b. Furthermore, in the case of p-doped (113)-grown QWs with C_s_ symmetry, interband transitions from hole states to electron states will follow Fermi’s golden rule and lead to net photocurrent involving both initial and final states. This is known as spin orientation-induced CPGE (described in Figure 3c). A sign inversion of current can be observed at the band edges by varying the light frequency [49].

#### 3.1.4. Coexistence of CPDE with CPGE Current

**Figure 4:**
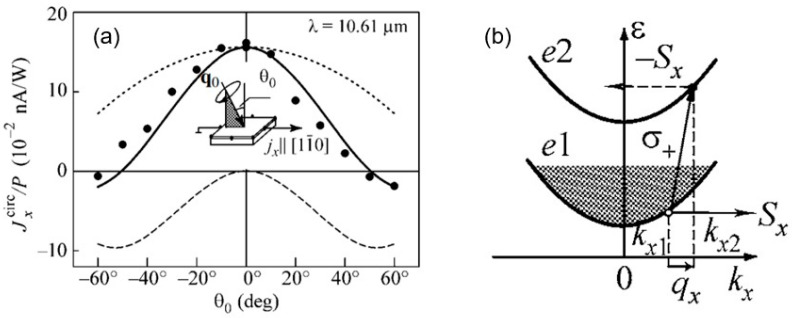
(**a**) Incidence angle dependence of normalized light helicity-dependent photocurrent obtained for (110)-oriented GaAs/Al_x_Ga_1-x_As with C_s_ symmetry under terahertz excitation. The solid dots represent the experimental data of the sample in the *x*-direction. The dotted, dashed, and solid curves are fitted to the phenomenological equation for the CPGE, the CPDE, and their combination, correspondingly. The inset shows the experimental geometry. (**b**) In addition to the CPGE described in Figure 3b, there are also intraband transitions (the CPDE, linear momentum transfer of photons shown by inclined up arrow) leading to spin polarization *S*_x_ (Adapted with permission from [50]).

Although the CPDE has been explained phenomenologically, its fundamental understanding was less studied until the work on the GaAs/AlGaAs QW system in the (100) growth direction with C_s_ symmetry [50], in which far infrared excitation on the sample generated a current $j_{\alpha }$ that had incidence angle dependence; strikingly at an incidence angle of approximately ±50°, the current changed sign. This was understood to be because there are two mechanisms in this system, the CPGE and CPDE; while the former has *sin*θ dependence, the latter has *sin*2θ, as seen in the upper dot curve and the lower dashed curve, respectively, as shown in Figure 4a. Microscopically, the PDE is an effect in which light momentum is transferred when carriers make an upward transition due to optical selection rules, and phonons are involved in the final states with absorption/emission processes (Figure 4b). This is valid for both the CPDE and the LPDE, while the CPDE can have spin selectivity similar to the CPGE.

### 3.2. Relation between the CPGE and the Rashba–Dresselhaus Effects

Spin photocurrents, such as the one due to the (magnetic field-dependent) spin-galvanic effect at normal incidence, were utilized in the discrimination of the Rashba and Dresselhaus effect since the *k*-cubic term has no influence on photocurrent [35]. Similarly, a spin photocurrent due to the (purely electric field of wave-dependent EM) CPGE at an oblique incidence can identify the Rashba term (*α*) and the Dresselhaus term (*β*) [51]. It is straightforward to understand the CPGE due to the Rashba effect since it is a surface or interface effect, while the bulk effect of Dresselhaus can also lead to the CPGE as there is interference between *α* and *β* terms resulting in anisotropy of splitting. In the 2D structure of the GaAs QW in the (001) growth direction possessing C_2v_ symmetry, under an oblique incidence of circularly polarized light, a CPGE current can be generated with photocurrent components due to the Rashba term as *j_R_* and the Dresselhaus term as *j_D_*. Their ratio strongly depends on the light propagation direction with respect to the crystal orientation [51] and can be used to determine the degree of inversion asymmetry, either SIA or BIA.

### 3.3. Photo-Induced Voltage

Spin photocurrents as a result of nonlinear photo-induced processes of the second order without applying a magnetic field lead to the identification of the Rashba/Dresselhaus effects and can originate as surface- and interface-related inverse Rashba–Edelstein effects. In addition to the spin photocurrents introduced above, it is also possible to resolve spin polarization through effects like the spin Hall effect, particularly via nonlinear photo-induced potential differentials between two points in a lateral structure. Here, we will first show the special case of photo-induced voltage by the spin Hall effect in a metal/semiconductor system with a large SOC. Then, we will give an example of photo-induced voltage in a metal thin film and briefly discuss possible outcomes.

#### 3.3.1. Photo-Induced Inverse Spin Hall Effect

**Figure 5:**
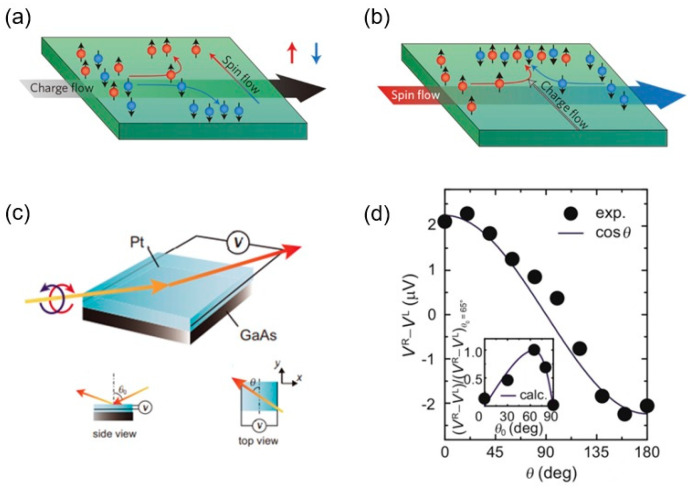
Description of the spin Hall effect (SHE) and the inverse SHE (ISHE), and experimental observation. (**a**) The SHE: a charge flow, orange and blue dots (spin-polarized electrons), induces a spin current perpendicular to the direction of the charge current due to spin–orbit force. (**b**) The ISHE, which is the reverse process of the mechanism in (**a**); a pure spin current makes both charges (with up and down spins) flow in the same direction, and a charge current will take place (From [56]). (**c**) A schematic illustration of the Pt/GaAs hybrid structure, in which photo-induced ISHE (PISHE) was observed. The incidence angle θ_0_ and the azimuthal angle θ were defined. (**d**) At θ_0_ = 65°, V^R^−V^L^ (PISHE) values were plotted as a function of θ, where V^R^ and V^L^ are voltage values for right- and left-circularly polarized radiation, respectively, for the sample shown in (**c**). The PISHE has a maximum value upon radiation along *y*-axis. The inset in (**d**) shows the θ_0_ dependence of V^R^−V^L^, which is maximized at ~θ_0_ = 65°, where the solid line is the calculated value of PISHE (From [58]).

Spintronics revolves around the problem of charge and spin interconversion. In addition to the aforementioned surface- and interface-dependent REE and IREE, there is also the spin Hall effect (SHE) [39,40,52] and the inverse SHE (ISHE) [53,54,55,56]. The SHE is described in Figure 5a. When a pure longitudinal charge current is injected into a semiconductor with strong SOC, electrons will experience a spin–orbit force, similar to the Lorentz force in ordinary Hall effects, and move in transverse directions (perpendicular to the direction of charge current), with spin polarization directions normal compared to the plane. The spin–orbit force acting on spin-polarized electrons will have an opposite sign for spin-up and spin-down electrons yielding a net spin current, known as the SHE. Conversely, when a pure longitudinal spin current (due to spin-polarized electrons) is injected into a semiconductor with strong SOC, as described in Figure 5b, the electrons with opposite spins will move in the same transverse direction, perpendicular to the spin current, and a net Hall charge current can be obtained, which is known as the ISHE.

The optical route is an effective method for the generation and manipulation of spin polarization via light polarization. The case of ISHE is a good example of this. A photocurrent can be generated upon circularly polarized radiation on a material system, such as a metal with strong SOC having an interface with a semiconductor, which is known as photo-induced ISHE (PISHE) [57]. As a spin-related nonlinear photocurrent, PISHE shares the same phenomenology with the CPGE phenomenology, with the only difference being that the PISHE takes place at high carrier densities and has a bulk-like effect. Microscopically, the spin–orbit force acting on electrons has spin selectivity, which makes them flow in the same direction as shown in Figure 5b, with an electromotive force of ***E***_ISHE_ transverse to spin current ***J***_s_ and spin polarization vector of the spin current ***Ƥ***, and efficiency D_ISHE_ as described in Equation (6),

(6) $$E_{\mathrm{ISHE}}=D_{\mathrm{ISHE}}\;J_{s}\times \;Ƥ.$$

The PISHE was discovered in metal Pt layer-deposited GaAs [57]. The system is described in Figure 5c. The role of the Pt layer was its large SOC, which is missing in GaAs. As it is a metal/semiconductor system, it is expected to show large photo-induced voltage. The sample plane is excited by polarized light (either σ+ or σ−) with an oblique incidence angle θ_0_, as shown in the bottom of Figure 5c. By varying the azimuthal angle (here defined as θ by [57]) from 0° transverse to 90° longitudinal, the photo-induced voltage for right- (V^R^) and left-circularly (V^L^) polarized excitation was measured using the lock-in technique. The difference between V^R^ and V^L^ corresponds to the electromotive force. In terms of the conversion of spin current to charge current, PISHE is similar to the IREE mentioned earlier.

One of the most important features of the PISHE is the angle of incidence dependence [58]. As can be seen in Figure 5d, there is a peak of the PISHE values at approximately 60°. This specific angle stems from the light diffraction through the material, which becomes crucial when bulk effects are considered. Normally, the phenomenology for the CPGE would dictate *sin*θ (peak at 90°) dependence and the CPDE would dictate *sin*2θ (peak at 45°). But in the case of the PISHE, this angle can be in between, depending on the material used as the SOC layer (Pt) and active layer (GaAs). Simply, the diffraction and bulk/interface spin effects determine the exact phenomenology and angle dependence of PISHE.

#### 3.3.2. Photo-Induced Voltage in Thin Metal Films

Nonlinear photo-induced voltage measurements are not limited to the PISHE photocurrent. In metals such as nanoporous gold (NPG) thin films [59], choosing a nanosecond laser with a 90° azimuthal angle from the electrodes (transverse geometry), at an oblique incidence, can generate incidence angle-dependent photo-induced voltage (PIV). Remarkably, for σ+ or σ− excitation, the PIV was completely opposite for 550 nm excitation. Although this result is similar in nature to the PISHE work on Pt/GaAs, its phenomenology was ascribed to the PDE. This is not a surprise as metals are expected to show PDE, although recent studies have questioned the sign of the PDE and the role of environmental effects [60]. The maximum value of PIV was obtained at an angle of incidence near 45° (*sin*2θ dependence) in [59], suggesting that the PIV was due to the CPDE, not PISHE or CPGE. Additionally, the PIV signal was linearly increasing from 510 nm up to 590 nm, implying there is a linear momentum transfer of light. Measurements performed in the longitudinal geometry showed LPDE features, while the CPDE was exclusively present in the transverse geometry and vanished in the longitudinal geometry. Similar to NPG PIV, plasmonic structures on gold films suggested CPDE [61,62,63], while the behavior of PIV with regular holes on a gold thin film (implying noncentrosymmetry) was also attributed to the transfer of light angular momentum, i.e., CPGE [62,63]. It is conceivable to observe CPDE features in high-carrier-density materials, while noncentrosymmetry can allow the coexistence of the CPGE (or PISHE) with CPDE.

## 4. Spintronic-Related Fundamental Properties of HOIPs

Among low-dimensional materials, the HOIP family has fascinating optoelectronic properties, such as external quantum efficiencies exceeding 20% for light-emitting diodes [64] and over 25% power conversion efficiencies for solar cells [65,66]. Robustness against environmental conditions and the stability of their chemical synthesis are progressing rapidly [66,67], which is promising for large-area production [68] and integrability into existing solar cell technology (such as silicon-based [69]). The main reason behind the outstanding optoelectronic properties is the flexibility in variation of their energy band gap by changing the organic part, which allows them to cover a wide spectral absorption range up to infrared. It also has the advantage of a long carrier diffusion distance and high mobility, in addition to the long recombination time. Here, the fundamentals of HOIP related to our focus will be visited in the context of spintronics.

### 4.1. Structural Properties of HOIPs

**Figure 6:**
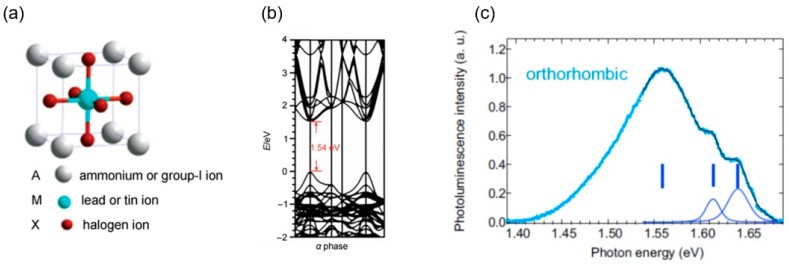
(**a**) Schematic diagram of the crystal structure of hybrid organic–inorganic perovskite (HOIP) and (**b**) calculated energy band structure of the most well-known HOIP, MAPbI_3_ or MAPI (from [70]) (**c**) Photoluminescence spectra of MAPI at 4 K for orthorhombic phase (the whole spectra data in cyan). The part of the spectra (in black) for energies above the indirect Rashba (~1.53 eV) to higher energies were fitted by three Gaussian (blue curves). Peak positions are indicated by vertical blue marks corresponding to transitions to Rashba-split states. (From [21]).

The unit cell of HOIP is like calcium titanate or an orthorhombic structure, as shown in Figure 6a, where A ions are in the eight top corner positions of the cubic crystal structure as monovalent cations, such as an organic methylammonium ion CH_3_NH_3_^+^, an organic formamidinium ion CH[NH_2_]_2_^+^, or an inorganic cesium ion (Cs^+^) as a group-I cation; M ions are in the body center of the cube as positive divalent metal ions, such as lead ion (Pb^2+^), tin ion (Sn^2+^), and germanium ion (Ge^2+^); and X ions are negative monovalent halogen ions, such as fluoride (F^−^), chloride (Cl^−^), bromide (Br^−^), and iodide ions (I^−^) [70]. The radii of the A-site ions must meet certain conditions and be able to populate a chalcogenide crystal. An M-site ion and six X-site ions form an MX_6_-type octahedral structure, and the radius of the A-site ion must be large enough to extend into that framework. The stability of the chalcogenide-type cubic structure is determined by the distance between the ions. Strong light absorption is a distinctive feature of chalcogenides. A typical example is methylammonium lead iodide CH_3_NH_3_PbI_3_ (MAPbI_3_), shortened to MAPI, which is a direct band gap material. It has the ability to absorb the full spectrum of visible light for all wavelengths. In the case of MAPI, there are three different structural phases as a function of temperature: orthorhombic below −113 °C, tetragonal from −113 to 57 °C, and cubic above 57 °C [71].

### 4.2. Rashba-Split States in MAPI Observed by Photoluminescence

One of the most important optical properties of HOIPs, particularly those that fall in the category of lead halide perovskites (LHPs), is that it is possible to vary the absorption, emission, and various other photo-induced processes by changing the organic part. This is a very attractive flexible feature and a subject of intensive research with rich literature. Here, we dwell on an exemplar LHP, MAPI, and its specific photoluminescence (PL) properties. In Figure 6c, the PL spectra exhibit several peaks due to optical transitions related to Rashba-split states for orthorhombic MAPI [21]. The lowest energy transitions are at ~1.53 eV, which are indirect Rashba transitions, and the energies above correspond to direct Rashba transitions (as fitted by three Gaussian curves in Figure 6c, with that at ~1.56 eV being quite weak). This corresponds to an orthorhombic phase at 4 K. At room temperature, as the phase changes to tetragonal, the lowest energy transitions move to ~1.64 eV, and direct Rashba transitions in PL may not be resolved; however, it can be resolved in a photocurrent.

### 4.3. Electrical Properties of Polycrystalline MAPI

Unlike the optical band gap, the transport gap of HOIPs, and particularly LHPs, is large for a direct gap material. In addition, the usual polycrystalline nature of HOIPs does not favor high intrinsic mobilities. However, there are advantages of such a polycrystalline structure of HOIPs due to A ions in a solar cell device structure. This can be understood in terms of its inorganic metal halide chalcogenide part, which has a 3D structure with high carrier mobility and a long carrier lifetime, unlike its organic part. Improvements in electrical properties rely on engineering between the chalcogenide structure and the organic complex, as well as their interfacial interactions and lattice distortions.

The family of HOIPs has inherited the issue of grain boundaries and trap density in electrical transport from the ionic nature of the organic part. Therefore, most electrical transport studies have focused on dielectric properties and *ac* ionic conductivity. To our knowledge, the smallest resistivity is ~ 70 kΩ·cm for MAPI [72] and ~30 kΩ·cm for MAPbBr_3_ (another member of the HOIP family) [73] at 350 °C. Similar to MAPI, in MAPbBr_3_, trap density and carrier mobilities can change with various crystal plane directions, which can expectedly halt their electrical transport performance and related charge carrier efficiencies; the highest dark (no light) current density can only reach 10 μA/cm^2^ [74]. The main obstacle for this is the organic nature of A ions in ABX3 of HOIPs, which refers to “ionotronics”, which can also affect their photoelectronic properties to some extent. More specifically, factors such as the size and electron affinity of A ions can reflect the electrical properties of HOIPs, as well as the crystallinity (single crystal or polycrystalline), energy band structure, and charge transfer rate. In addition, it was found that doping A ions can effectively improve the stability and performance of HOIPs, providing new possibilities for their applications in optoelectronics [75]. For the above reasons, most studies on HOIPs alone are capacitance, *ac* conductivity, and photocurrent studies, in which single crystals of HOIP like MAPI can yield photocurrent values of a few nanoamperes.

### 4.4. Spintronics Based on LHPs

**Figure 7:**
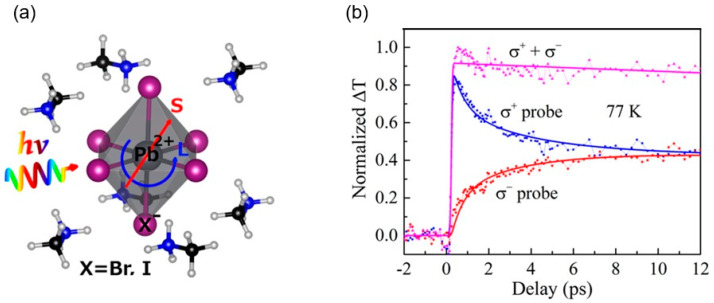
(**a**) Local perturbations in the CH_3_NH_3_PbX_3_/TiO_2_ cubic lattice (from [77]). (**b**) Normalized circular pump–probe decay transients with 19 μJ/cm^2^ σ+ pump and σ+ probe (blue), σ− probe (red), and their total (magenta) at 77 K. The experimental data are globally fitted using time-dependence function and the results show that the σ+ and σ− relaxations occur in a time scale (~7 ps) much shorter than the carrier recombination lifetime (from [78]).

Thanks to strong SOC, most HOIPs, particularly LHPs, have spin-dependent properties, which can cause them to be spintronic- or spin–orbitronic-related materials. The previously mentioned Rashba effect is prominent in LHP due to the SOC of heavy elements (Pb) in its inorganic part, which was susceptible to magnetic field effects. Magneto-optical studies showed spin-mixing of the photogenerated electron–hole pairs in the conduction band with different *g*-factors [76]. In fact, the strong SOC in LHPs and large splitting of conduction bands, rather than the usual splitting of the valence bands as in conventional semiconductors with SOC, was first expected and explained in terms of direct and isotropic optical transitions with spin–orbit split-off bands related to the triply degenerated conduction band, being only slightly affected by local distortions of the lattice [77], as shown in Figure 7a. Thanks to this robustness, the valence band offset of LHPs aptly matches TiO_2_ (as a conventional transport layer) in terms of band alignment for the purpose of driving the electrons toward the electrode. This could make the design of colorful solar cells with enhanced light conversion efficiency possible [77]. It was also shown that MAPI can reach a 7 ps spin lifetime in Faraday rotation for circularly polarized excitation (Figure 7b) [78]. These two examples showcase the benefits of the strong SOC of LHPs.

**Figure 8:**
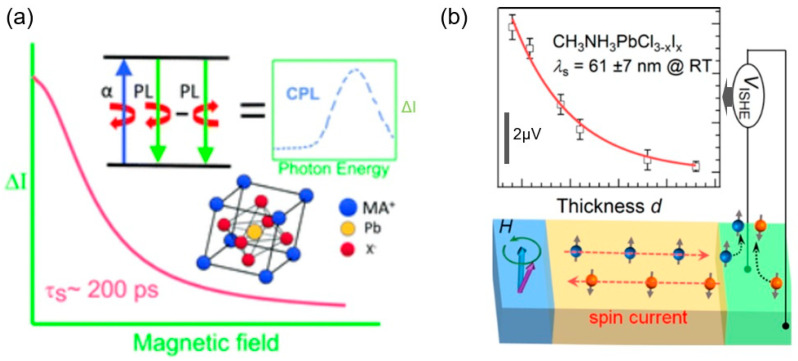
(**a**) The dependence of luminescence intensity ($\Delta I=\mathrm{PL}\left(\sigma^{-}\right)-\mathrm{PL}\left(\sigma^{+}\right)$) on the magnetic field (with Δ*I* being in arbitrary units); the top illustration represents the principle of circularly polarized photoluminescence (CPL), derived from partial CPL produced under circularly polarized light excitation at 77 K for a perovskite consisting of methylammonium ion MA+, Pb, and halide X^−^. Here, *τ*_s_ is the spin lifetime (From [80]). (**b**) The plot shows the dependence of the ISHE voltage (V_ISHE_) as a function of perovskite active layer thickness *d* and the schematic below describes the device made out of CH_3_NH_3_PbCl_3−x_I_x_ film and the spin current generation. The fitting curve (red) is an exponential decay curve, from which the spin diffusion length $\lambda_{s}$ was extracted (from [81]).

However, more importantly, the motivation of spin studies on LHPs was not limited to photovoltaic enhancements only. The change in the symmetry at the bottom of the conduction band with SOC changes the optical selection involving electron–phonon processes, which directed attention to the Rashba and Dresselhaus terms [44], as demonstrated by angle-resolved photoemission spectroscopy (ARPES) [79]. Obviously, this could be thought of as spin manipulation and spin transport in the context of spintronics if a spin injection is possible using ferromagnetic film or contact with LHPs. This started the Hanle effect studies in LHPs, and a spin lifetime *τ*_s_ of 240 ps [80] was obtained, as shown in Figure 8a. In another work, an IREE coherence length of up to ~30 pm and 190 ps was observed for samples with NiFe on MAPbBr_3_ by microwave Hanle measurements [47].

Concerning ferromagnets on LHP systems, there are still several debates. In [81], spin pumping was used to demonstrate the inverse spin Hall effect (ISHE), with a spin diffusion length as long as 61 ± 7 nm at room temperature, as shown in Figure 8b. This was explained in terms of spin transmission through domain boundaries, where local Rashba spin splitting is in effect, meaning the overall bulk effects expected in SHE are a collection of domain boundary/interface effects, implying that spin-to-charge conversion could have a big impact on optoelectronic properties. In fact, this was previously expected by the diffusive transport model and calculated spin densities [82].

## 5. Nonlinear Photocurrents in LHPs

In recent years, with the continuous pursuit of solar energy efficiency, LHP-based materials have attracted much attention, including their spin properties. As they are noncentrosymmetric materials, the phenomenology of nonlinear optical processes explained previously can be applied to LHPs, allowing photocurrents due to the PGE and PDE effects, such as in the case of MAPI with C_4v_ symmetry [45]. Nonlinear photocurrents in LHPs can be categorized into two groups: single-crystal and polycrystalline structures.

### 5.1. Single-Crystal LHP Nonlinear Photocurrent

The structural properties of LHPs are an important research topic, as structural properties can affect various device performances. Therefore, there have been several attempts to understand phase changes from orthorhombic (at low temperatures) to tetragonal (at room temperature) [21]. It was found that nonlinear photocurrents can be useful in explaining the structural effects. From a phenomenological approach, this can be understood in terms of centrosymmetry and crystallographic orientations. Nonlinear photocurrents, particularly spin photocurrents such as the one due to the CPGE, can be used to study the structural information in LHPs.

**Figure 9:**
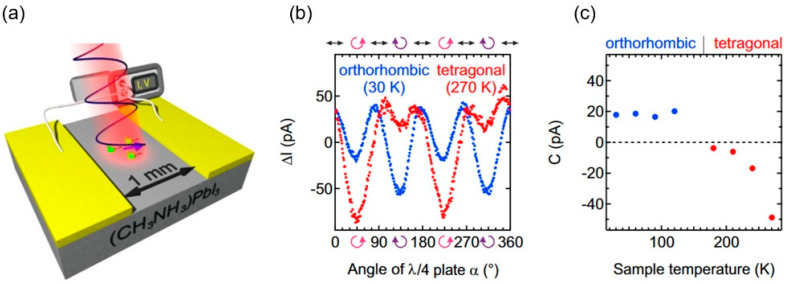
(**a**) (CH_3_NH_3_)PbI_3_ single-crystal sample with gold contacts (channel width: 1 mm) is illuminated at oblique incidence *θ* with tunable photon energy close to the band gap. (**b**) Photocurrent data recorded as a function of angle of photon polarization (α) for two different structural phases, which was followed by fitting the data to Equation (5) and determining the C (circular photocurrent values). (**c**) The extracted C values were plotted as a function of temperature (experimental parameters: photon energy of 1.52 eV for the tetragonal and 1.61 eV for the orthorhombic phase; *θ* = 40°; laser power of 40 μW) (from [21]).

The first attempt to study the CPGE on LHPs was on single-crystal samples, such as the one in Figure 9a. In a MAPI sample, it is possible to obtain a single crystal on a mm scale, and as shown in Figure 9b, it was possible to obtain a nonlinear photocurrent of ~100 pA, upon light helicity, at room temperature [21]. Microscopically, this photocurrent was explained by transitions just below the direct gap as a result of the Rashba effect and was labeled a circular photocurrent (C, which should be the same constant of $C_{1}$ in Equation (5) or a combination of $C_{1}$ and $C_{2}$); however, as we explained in Rashba-split states in MAPI by PL, the CPGE or the CPDE being the origin of this photocurrent is not straightforward. For the purpose of identification, temperature-dependent measurements were performed. At low temperatures, the amplitude of this photocurrent decreased and changed signs (Figure 9c), which implied dependence on the band edge, and this was the first attribution of the CPGE as a measure of structural fluctuations.

**Figure 10:**
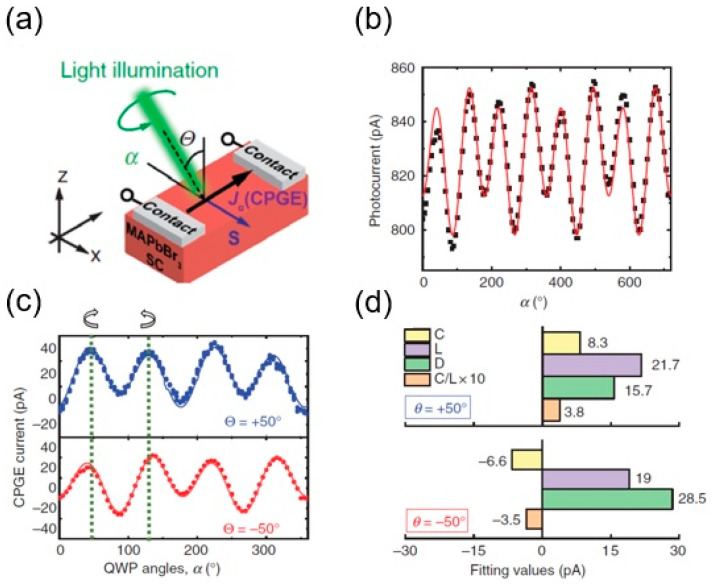
(**a**) A sketch of nonlinear photocurrent experimental setup based on MAPbBr_3_ SC, where *θ*, *α*, *S,* and *J*_c_ (CPGE) represent the angle of incidence, the angle of light polarization, the spin orientation, and the pure spin current due to circular photogalvanic effect—CPGE. (**b**) The obtained photocurrent response as a function of the QWP rotation angle or angle of photon polarization (*α*) (from 0° to 720°, repeating itself in cycles of 360°). The data were fitted to *J*_c_ = C *sin* (2α) + L *sin* (4α) + D, where L is a combination of L_1_ and L_2_, and C is the CPGE contribution. (**c**) The extracted CPGE values as function of *α*, at two impinging angles: *θ* = 50° (blue symbols), *θ* = 50° (red symbols), and fitting results are compared in (**d**) (from [46]).

There has been increasing interest in nonlinear photocurrents on single-crystal LHPs and the direct/indirect Rashba transitions. A more sophisticated LHP with indirect tail states, MAPbBr_3_, has been studied by nonlinear photocurrent measurements to determine the role of broken inversion symmetry (due to specific surface termination), which was not observed in SHG and ARPES measurements. The significance of the nonlinear photocurrent in these samples concerned a fundamental understanding of the crystallographic structure of MAPbBr_3_ at room temperature, which could have local noncentrosymmetry with global centrosymmetry (also labeled pseudo-centrosymmetry) [83]. For the measurement setup depicted in Figure 10a, a nonlinear photocurrent of ~800 pA was observed, as shown in Figure 10b, where *α* was defined as the angle of photon polarization and the data repeated itself in cycles of 360°. The extracted CPGE part of the nonlinear photocurrent was plotted in Figure 10c as a function of the angle of photon polarization. There was a difference between +45° and −45° incidence angles; the C component has an opposite sign with approximately the same amplitude (Figure 10d). This raises the question of whether the source of the circular photocurrent is only due to the CPGE or whether there could be CPDE effects too.

**Figure 11:**
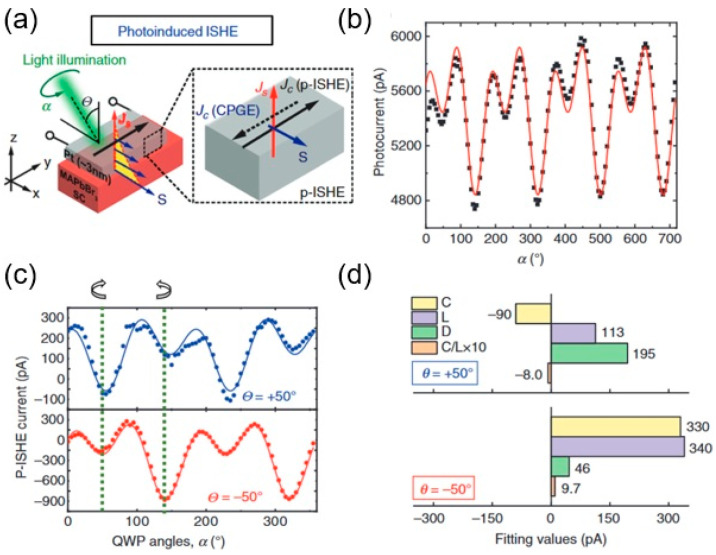
Photo-induced inverse spin Hall effect (PISHE) and circular photogalvanic effect (CPGE) measurements (**a**) Sample and measurement description on the left; and on the right, mechanism of PISHE (*J*_c —PISHE_) taking place at the MAPbBr_3_SC/Pt interface together with the CPGE (*J*_c —CPGE_) is explained together with *J*_s_ spin pumping. (**b**) The angle of photon polarization (*α*)-dependent photocurrent data (from 0° to 720°, repeating itself in cycles of 360°) was fitted to *J*_c_ = C *sin* (2α) + L *sin* (4α) + D, where C consists of PISHE and CPGE (**c**) The extracted PISHE values as function of *α*, at two impinging angles: *θ* = 50° (blue symbols), *θ* = 50° (red symbols) and fitting results were compared in (**d**) (from [46]).

In the same work [46], in order to study the effect of external SOC, a 3 nm Pt layer was used on the sample (Figure 11a), similar to the work on Pt/GaAs [58]. When the sample was excited by circularly polarized light, the nonlinear photocurrent increased 10 times compared to that without a Pt layer (Figure 11b). Also, there was another sign change between incidence angles of +50° and −50°, but also an amplitude difference (Figure 11c,d), which was attributed to the coexistence of surface/interface current due to the CPGE, as well as the other spin-to-charge conversion mechanism PISHE. These data suggested that there are still several open questions about the origin and source of these currents. The coexistence of two spin-to-charge conversion mechanisms, PISHE and IREE, may be more complicated when taking both surface/interface and bulk effects into account.

### 5.2. Polycrystalline LHP Nonlinear Photocurrent

**Figure 12:**
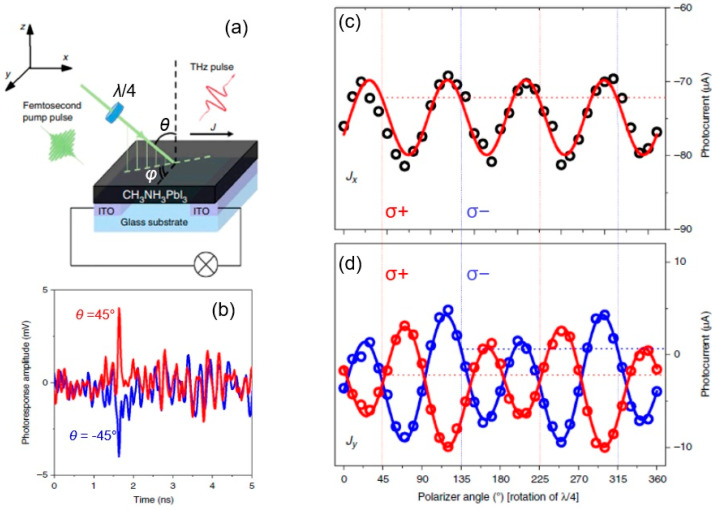
Polarization-dependent photocurrent in unbiased polycrystalline MAPI sample at room temperature. (**a**) Measurement configuration and (**b**) temporal profiles of photocurrent response measured at different incidence angles *θ* (*θ* = 45°: red; *θ* = −45°: blue) in the transversal (azimuthal angle *φ* = 90°) geometry of the experiment. (**c**,**d**) Excitation beam polarization dependence of longitudinal (*J*_x_, *φ* = 0°) and transversal (*J*_y_, *φ* = 90°) components of photocurrent response measured at *θ* = 45°. The excitation beam polarization state is controlled by rotating a QWP (λ/4) with 5° steps; and 45° (135°) and 225° (315°) correspond to left- (right-) circular polarization. The open circles are the experimental data. The fitting curves are the phenomenological fitting function, *J* = *J*_0_ + C *sin* (2α) + L_1_ *cos* (4α) + L_2_ *sin* (4α) (from [84]).

Polycrystalline LHPs have the advantage of facile growth and large-scale production, which are important for solar cell applications. Furthermore, it can offer a rich spin-dependent flexible optoelectronic platform. In nonlinear photocurrent work on MAPI, terahertz pulses (see Figure 12a) were used to study the PGE and PDE effects [84]. In this work, as used in some papers, the bulk photovoltaic effect term is used as an alternative name for the PGE. The CPGE was referred to as an injection current, and the LPGE was referred to as the shift current [85]. The observed ultrafast photocurrent had a sign change from angles of incidence of +45° to −45° in the transverse direction (Figure 12b), which was attributed to the existence of the PDE since the current changed signs by changing the propagation direction, unlike the shift current sign, which remains the same. In longitudinal geometry, the angle of photon polarization dependence for incidence angles of +45° and −45° was the same, implying only one effect is in action (Figure 12c); while, in transverse geometry, for incidence angles of +45° and −45°, the angle of photon polarization dependence was completely opposite (Figure 12d), indicating the existence of the PDE photocurrent as well. Therefore, polycrystalline LHP samples could be a good platform to study the PGE- and PDE-related nonlinear photo-response.

### 5.3. Spin-to-Charge Conversion in LHPs

Most nonlinear photo-induced studies on MAPI were performed using single-crystal samples. Similar to previous nonlinear photocurrents of other materials, lateral device geometry was employed to study the CPGE current in LHPs, which was later followed by the inclusion of a magnetic metal layer on LHPs to study the PISHE current. In either case, spin-to-charge conversion, either like surface/interface-dominated IREE or bulk-related PISHE, was reported; and the identification and separation of both conversion mechanisms was defined based on interconversion efficiency, which can be expressed, for PISHE, in terms of the Hall angle *θ*_ISHE_ = *j*_3D_^c^/*j*_3D_^s^ [46], where *j*_3D_^c^ and *j*_3D_^s^ are the 3D current density of charge and spin, respectively, and for IREE, in terms of the IREE length (λ_IREE_ = *j*_2D_^c^/*j*_3D_^s^ = α_R_ τ_s_/ħ), where *j*_2D_^c^ and *j*_3D_^s^ are the 2D current density of charge and 3D current density of spin, respectively, *α*_R_ is the Rashba coefficient, and *τ*_s_ is the effective spin-momentum scattering time at the Fermi surface. Recently, these two effects were contrasted in [46] in the sense that a spin current is driven via a magnetic layer on LHPs. This necessitates the application of a magnetic field to observe both PISHE and IREE, which may separate the 3D bulk from the 2D surface/interface influence. However, a magnetic field is not the sole condition to obtain either effect. As was shown in [86,87,88], a polarized light field such as left- and right circularly polarized light can satisfy the condition in the separation of 2D from 3D. On the other hand, a relatively more important criterion is the carrier density. In recent DFT work regarding the IREE [22], it was shown that 2D spin density can increase as 2D charge density passes the 10^12^ cm^−2^ mark, while the spin polarization fraction σ continues to drop. Meanwhile, as 2D current density *J*_2D_ increases, spin-to-current density will decrease. This suggests that the IREE can be indeed comparable to PISHE depending on carrier densities, in addition to material, measurement, and light-impinging geometries.

## 6. Summary and Future Prospects

Research on perovskites is quite broad, vibrant, and interdisciplinary. There are great publications at the intersection of various fields. In this comprehensive article, we aimed to bring attention to an emerging branch of research on one of the nonlinear photoinduced processes, which can also be called nonlinear photocurrents, namely, lead halide perovskites—LHPs. Nonlinear photocurrents related to spin-related phenomena in LHPs, exclusively those with organic monovalent cations, have been reviewed in terms of the Rashba effect, polarized light–matter interaction phenomenology, and governing microscopic mechanisms with comparison to other semiconductor, metal, and hybrid systems. With promising optoelectronic, spintronic, and spin–orbitronic properties, LHPs with organic monovalent cations offer so much with their flexible features, such as band gap and SOC tunability. Therefore, there has been a surge of interest in studying LHPs and other HOIPs in the frame of PISHE and/or IREE spin-to-charge conversion, which are among the main tools used in the modulation of opto-spintronic device properties.

The research on nonlinear photocurrents in LHPs not only promises advances in optoelectronic devices for spintronics. It is also capable of contributing to a fundamental understanding of spin–orbit coupling (SOC) in semiconductor systems and dimensionality effects on the photophysical properties of organic semiconductors, which correlate to some extent. For instance, recently, it was expected that SOC can retard nonradiative recombination processes in methylammonium lead triiodide perovskites [89]. Also, the bulk Rashba effect was revealed in similar samples [86]. Regarding the Rashba effect, research on strong SOC materials possessing SIA or BIA/IIA is quite active. The interface between LHPs and materials with weak and strong SOC should be elaborated. As was shown in [46], the interface could play an important role in the nature of the Rashba effect. At the same time, “spin interfaces” such as LHPs with ferromagnetic materials require more attention for advances in molecular spintronics [90]. The Rashba and Dresselhaus SOC strengths can be also further studied using nonlinear photocurrent measurements in LHPs with varying sizes and types of organic parts, similar to recent work [91] in which a change in crystal structure with lead vacancies was studied by the CPGE photocurrent, emphasizing the role of defects.

From a broader perspective, beyond the pioneering work of the use of CPGE as an indication of structural fluctuations in MAPI [21], there is a need for systematic nonlinear photocurrent investigations on LHPs. This can be performed in a similar way to the PL studies on solar cell devices [92], with a particular focus on stability issues—a fundamental problem to address in order to move towards solar cell commercialization of perovskites [93]. For instance, the CPGE could be another measure of stability/durability in indoor–outdoor performances of p–i–n perovskite solar cells [94], as well as hole transport material-free ones [95]. In particular, for the latter case, the modification of interfaces and defect control could be performed by CPGE and PISHE analysis depending on the materials used. In addition to [94], tandem solar cells based on perovskites [69,96,97] show promise against stability challenges; and, as scrutinized in this review, there are several possibilities to utilize nonlinear photocurrents in terms of both bulk and surface effects in tandem solar cell devices. Furthermore, the use of metallic nanoparticles in perovskite solar cells, a promising method for large-scale reliable production, can be subject to enhanced second-order nonlinear photocurrents to be used as a characterization tool, beyond the plasmonic photovoltaic effect [98]. At this point, we need to note that in terms of manufacturing, graphene (a well-known CPGE material) has already been used together with perovskites in solar panels, providing high chemical and panel stability [99].

Another important aspect of nonlinear photocurrents on LHPs with organic monovalent cations is that it can open a new chapter in spin-to-charge current interconversion in organic spintronics. As one of the main driving forces of spintronics, spin-to-charge conversion has been found to be utilized as a result of broken time-reversal symmetries as well as broken inversion symmetry, including chiral organic materials (see review [100]). As was demonstrated in [46,83], nonlinear photocurrents due to IREE- and PISHE-based spin-to-charge conversion can compete and their sign is opposite. In order to observe PISHE, there is the necessity for a ferromagnet/LHP interface. Various measurement geometries and samples with chiral point group symmetries (such as C_3_) should be further considered for nonlinear photocurrent measurements for the interplay between PISHE and IREE. Moreover, based on promising CPGE results for 2D chiral HOIPs [101], the relationship between symmetry structures in LHPs (such as chiral layers) and spin-to-charge conversion should be studied since the expectation of charge-to-spin and Dresselhaus-type spin textures was reported in telluride tetrahedral clusters [102].

On a final note, it is worth remembering that LHPs with organic monovalent cations are the intersection point of organic spintronics and perovskite physics with various spin-dependent properties [103]. These excellent solar cell materials are expected to yield large spin-dependent responses, even to unpolarized light [82], and contribute to photovoltaic efficiency via the conversion of photogenerated singlet excitons to nonradiative triplet excitons using properly adjusted chiral light [104]. In the guidance of recent studies on the light helicity dependence of photocurrents and photovoltages on organic solar cells [105] and light polarization-dependent third-generation perovskite solar cells [84], nonlinear photocurrents in LHPs shine like a north star.

## Data Availability

Not applicable.
